# Treatment of Ammonium-Rich Digestate from Methane Fermentation Using Aerobic Granular Sludge

**DOI:** 10.1007/s11270-018-3887-x

**Published:** 2018-07-09

**Authors:** Piotr Świątczak, Agnieszka Cydzik-Kwiatkowska

**Affiliations:** Department of Environmental Biotechnology, University of Warmia and Mazury in Olsztyn, Słoneczna 45 G, 10-709 Olsztyn, Poland

**Keywords:** Digestate treatment, Aerobic granular sludge, Nitrogen removal, *Thauera* sp.

## Abstract

Digestate produced by cofermentation of agricultural waste and manure can be difficult to dispose of because its high ammonium content impedes its use in agriculture due to generation of odor and overfertilization. This study investigated the possibility of treating such nitrogen-rich digestate with aerobic granular sludge depending on the nitrogen load in the reactor. At nitrogen loading rate of 1.0 g TN/(L·day), the nitrogen removal efficiency was high (64.9 ± 9.8%), ammonium nitrogen was completely oxidized, and nitrate was the main nitrification product. At nitrogen loading rate of 3.4 g TN/(L·day), ammonium oxidization was still good (93.6 ± 2.0%), but the percentage of partial nitrification was high (over 68%) and nitrogen removal efficiency worsened to 30.2 ± 2.6%. Despite this, the overall amount of nitrogen removed was 0.86 g TN/(L·day) and was over nearly two times higher than at the lower nitrogen loading rate. At both nitrogen loading rates, in the effluent nitrogen in a form of suspended solids predominated. To diminish the overall N loading in the effluent, treatment is therefore recommended enabling removal of solids, e.g., microfiltration, should be applied, or the digestate should be separated into solid and liquid phases, and only the liquid fraction should be subjected to biological treatment. At high N load in aerobic granules, a very versatile community of N-metabolizing microorganisms was present. More than 50% of all bacteria in aerobic granules were able to metabolize nitrogen, and the predominant genera (35%) was *Thauera*, which indicated that stable ammonium removal was achieved mostly as a result of heterotrophic nitrification.

## Introduction

The final product of anaerobic digestion, in addition to methane-rich biogas, is digestate. The amount of agricultural biogas plants in Europe is growing (currently about 14,500 plants), and they are struggling with the problem of managing digestate. Therefore, the sustainability of biogas production depends greatly on the effective disposal of the produced digestate. The fact the EU Waste Directive considers digestate to be waste does not make the situation easier (Wawrzyniak and Zbytek 2015).

The composition and quality of digestate is highly dependent on the composition and quality of the feedstock used. The quality of digestate can be also improved by improving the fermentation efficiency by applying, e.g., feedstock ultrasonication. Ultrasonication destroys complex organic molecules that have not undergone biological hydrolysis that results in an increase in the amount of biogas produced up to 30% in relation to non-ultrasonified substrate (Boni et al. 2016). In contrast to well-defined biomasses such as digested sludge, the properties of digestate from fermentation of waste from the agricultural and food industry are not well known. Digestate usually has very good fertilizing properties because of its high content of nutrients (N, P, K) in available forms. Because of this, digestate appears to be a good candidate to replace inorganic fertilizers, high-quality compost, and soil improvers, providing benefits for society in general and for the environment in particular, as well as helping to preserve limited natural resources such as fossil resources of mineral phosphorus. Digestate is usually biologically stable and partially hygienized (Tambone et al. 2010). If used for fertilization, digestate application must be consistent with legislative documents that define the nitrogen load on farmland (e.g., regulated inside the EU by the EU nitrate-directive (91/676/EEC nitrate)). The simplest way to use digestate as fertilizer is to apply the “whole digestate” to crop fields without any further treatment. In such cases, the soil must be able to accept a very large charge of nutrients, which is a rare situation. An accumulation of biogas plants in a small area and regular fertilization with digestate can lead to an overdose of digestate and consequent soil erosion. As a result, water-air interactions in the soil can be affected, and groundwater can be polluted. The use of digestate as a fertilizer may also be limited in winter (so that sufficient digestate storage capacity needs to be established) as well as in areas threatened by excessive eutrophication (lagoons, river basins).

The agricultural utilization of digestate can be limited by the presence of high concentrations of nitrogen compounds, which is a common result of cofermentation with liquid manure. In EU, the maximum amount of animal manure that can be applied to land every year is equivalent to 170 kg organic N/ha (Good Agricultural Practice for the Protection of Waters 2009, S.I. No.101 of 2009).

The use of unprocessed digestate in the environment can cause approximately 70% of the nitrogen to be emitted as NH_3_ (Pötsch et al. 2004; Bauer et al. 2009). In such cases, other solutions for digestate processing and utilization must be used, such as physicochemical or biological methods. When looking for purification methods for digestate, we should also focus on solutions that apart from solving the problem of digestate also give economic benefits. Examples of such measures may be the precipitation of phosphorus in the form of struvite or the recovery of ammonium nitrogen for the production of fertilizers (Vaneeckhaute et al. 2017).

A chemical method that can be used for digestate treatment is stripping. Limoli et al. (2016) tested CaO, NaOH, and H_2_O_2_ as agents for ammonia stripping. After incorporation of the appropriate compound, the reactor contents were mixed. Ammonia removal efficiency was best (51.6%) with NaOH at pH 12 after 40-min stirring. Another interesting method of nitrogen removal is electrocoagulation. Mores et al. (2016) have shown that inserting electrodes under constant voltage into the digestate removed total nitrogen with 10% efficiency and organic carbon with 70% efficiency. An effective ammonium removal at the level of 95% was also ensured by a vacuum thermal stripping (Ukwuani and Tao 2016).

Biological methods are cheaper than physicochemical treatment, but their main limitation is the resistance of biomass to high pollutant loads in the influent to the reactor. Activated sludge technology is the most common and widely used method of biological treatment of urban and industrial wastewater (Klimiuk and Łebkowska 2008); however, it is gradually being replaced by aerobic granular sludge technology. The concentration of biomass in aerobic granular sludge reactors can be two to three times higher than the concentration of biomass in activated sludge systems, which results in higher treatment efficiency and lower operational costs. Biological treatment based on aerobic granular sludge can efficiently remove nitrogen from high nitrogen wastewater with a low COD/N ratio and a high N load, such as landfill leachate (Mieczkowski et al. 2016) or digestate from dewatering of digested sludge (Cydzik-Kwiatkowska et al. 2013). However, the applicability of granular sludge technology to the treatment of digestate characterized by a high COD/N ratio and high organic and nitrogen loads has not been tested. Aerobic granules cope well with high organic loading, which favors the stability of their granules (Wang et al. 2008), and the very long sludge age in granular sludge systems should ensure good conditions for the growth of nitrifiers in multilayer granule structures.

The aim of this study was to investigate nitrogen removal efficiency using aerobic granular sludge technology from digestate with high nitrogen and organics loads. The microbial structure of the aerobic granular biomass exposed to high pollutant loads was analyzed to conclude about the groups of bacteria and metabolic pathways responsible for nitrogen removal.

## Materials and Methods

### Substrate

The digestate was taken from an agricultural biogas plant codigesting liquid manure and corn silage (at 7:3 volumetric ratio) in wet fermentation (the agricultural biogas plant in Łęguty, Poland). The characteristics of the digestate were as follows: 33 g TSS/L, 6.4 g TKN/L, 1.8 g N-NH_4_^+^/L, 0.33 g TP/L, 19.1 g COD/L (11.7 g SCOD/L), and a BOD_5_/COD ratio of about 1:1. The alkalinity and pH were 19.5 mval/L and 8.7, respectively. The substrate was stored at 4 °C.

### GSBR Operation

The experiment was carried out in two sequencing batch reactors with a working volume of 7 L operated at a cycle length of 8 h. The volumetric exchange rate was 35%. The aeration rate in the reactors was 4 L/min. The feeding, settling, and discharging phases in the reactor cycle lasted for 5 min. Very high concentrations of pollutants in the digestate made it necessary to dilute the digestate prior to introduction to the biological reactor. The reactors were fed with a mixture of digestate and rain water and operated at nitrogen loading rates of 1.0 g TN/(L·day) (103 cycles) and 3.4 g TN/(L·day) (41 cycles). At 1.0 g TN/(L·day), biomass concentration was 11.1 ± 0.0 g MLSS/L (8.1 ± 0.0 g MLVSS/L) while at 3.4 g TN/(L·day), biomass concentration was 13.0 ± 0.3 g MLSS/L (6.5 ± 0.1 g MLVSS/L). The pH in the reactors varied between 9.0 and 9.5. In the initial hours of the cycle, DO was below 0.5 mg/L due to intensive usage of oxygen for organics and ammonium oxidation but increased from the fourth hour of the cycle and stabilized at 6 mg/L until the end of the cycle.

### Physicochemical Measurements

The concentrations of total nitrogen, total phosphorus (HACH tests), ammonium nitrogen, orthophosphates, and COD (according to APHA 1992), as well as the pH and alkalinity (TitroLine Easy), were measured in the reactor influent and effluent. Additionally, concentrations of oxidized forms of nitrogen were measured in the effluent according to APHA (1992). The concentration of total suspended solids (TSS) in the influent and effluent, and the concentrations of mixed liquor suspended solids (MLSS) and mixed liquor volatile suspended solids (MLVSS) in the reactor and the sludge volume index (SVI) after 30 min of settling were measured according to APHA (1992). The size of granules was estimated by a wet sieving procedure as described in Cydzik-Kwiatkowska et al. (2009). Changes in oxygen concentration in the reactor were measured using a ProOdo probe (YSI Environmental).

### Calculations

The sludge yield coefficient (*Y*) was calculated according to Klimiuk and Kulikowska (2005). The calculation of free ammonia (FA) concentration at the beginning of the reactor cycle was performed as described by Ford et al. (1980). At the end of operation of the reactor at nitrogen loading rate of 1.0 g TN/(L·day) (full ammonium removal), pollutant concentrations were also measured during the reactor cycle to examine the kinetics of removal of organics, nitrogen and phosphorus by the aerobic granular sludge. Removal of orthophosphates and COD followed first-order kinetics, while ammonium removal followed zero-order kinetics.

To calculate nitrification efficiency, the concentration of the oxidized nitrogen forms in the reactor effluent was divided by the concentration of TKN at the beginning of the cycle less the N used for biomass synthesis. For denitrification efficiency, the concentration of N reduced in the reactor cycle was divided by the concentration of all oxidized nitrogen forms. The average efficiencies of pollutant removal were calculated from the last six measurements performed in the period of stable reactor operation.

### Next-Generation Sequencing

Next-generation sequencing was used to analyze microbiota of digester and aerobic granules from the reactor operated at nitrogen loading rate of 1.0 g TN/(L·day) (full ammonium removal). DNA isolation was done using a FastDNA® SPIN Kit for Soil (MP Biomedicals). The purity and concentration of the isolated DNA was measured with a Lite NanoDrop spectrometer (Thermo Scientific). A 515F/806 (GTGCCAGCMGCCGCGGTAA/GGACTACHVGGGTWTCTAAT) universal primer set targeting Bacterial and Archaeal 16S rDNA gene was used. The amplicons were sequenced using the MiSeq Illumina platform in Research and Testing Laboratory (USA). Over 43 thousand full sequences were obtained.

Sequences were analyzed bioinformatically as described in Świątczak et al. (2017). In short, sequences were clustered into operational taxonomic units (OTUs) using USEARCH global alignment (Edgar 2010). To query FASTA formatted files with seed sequences for each cluster against a database of NCBI-derived sequences, a .NET algorithm that utilizes BLASTN+ (www.krakenblast.com) was used. Sequences were aligned by Infernal (Nawrocki and Eddy 2013), clustered by Complete Linkeage Clustering using modules of the RDPipeline (http://rdp.cme.msu.edu/) and assigned to phylotype clusters at five cutoff levels: 1, 3, 5, 7, and 10%. Rarefaction analysis, the Evenness index, and the Shannon-Wiener index of diversity (H′) (all sequences were taken for the analysis) were calculated using RDP modules. The samples were characterized by similar amounts of sequences and were obtained in the same run, therefore, to avoid loss of data, the data were not normalized.

The sequences have been deposited in the NCBI Sequence Read Archive (SRA) within BioProject PRJNA407423 as the experiment entitled “Biological treatment of digestate” (Accession: SRX3189900, SRX3189903).

### Statistics

The technological results were analyzed with Statistica 12.5 (StatSoft). To compare the two samples, a *t* test for independent samples was used after normality and homogeneity of variance were confirmed with the Shapiro-Wilk test and Levene’s test. In cases of non-normal distribution, the non-parametric Kruskall-Wallis test was used. With all tests, *p* < 0.05 was considered significant.

## Results and Discussion

Digestate from agricultural biogas plants contains a large amount of nitrogen, phosphorus and potassium in forms readily available to crops and is characterized by higher microbiological and hygienic stability than unprocessed organic fertilizers (Tambone et al. 2010; Alburquerque et al. 2012a; Alburquerque et al. 2012b). Digestate may be a good alternative to inorganic fertilizers, but the NH_4_^+^-N content can pose a serious environmental problem. In the present study, aerobic granular sludge technology turned out to be an efficient solution for full oxidation of the ammonium nitrogen present in the digestate.

**Fig. 1:**
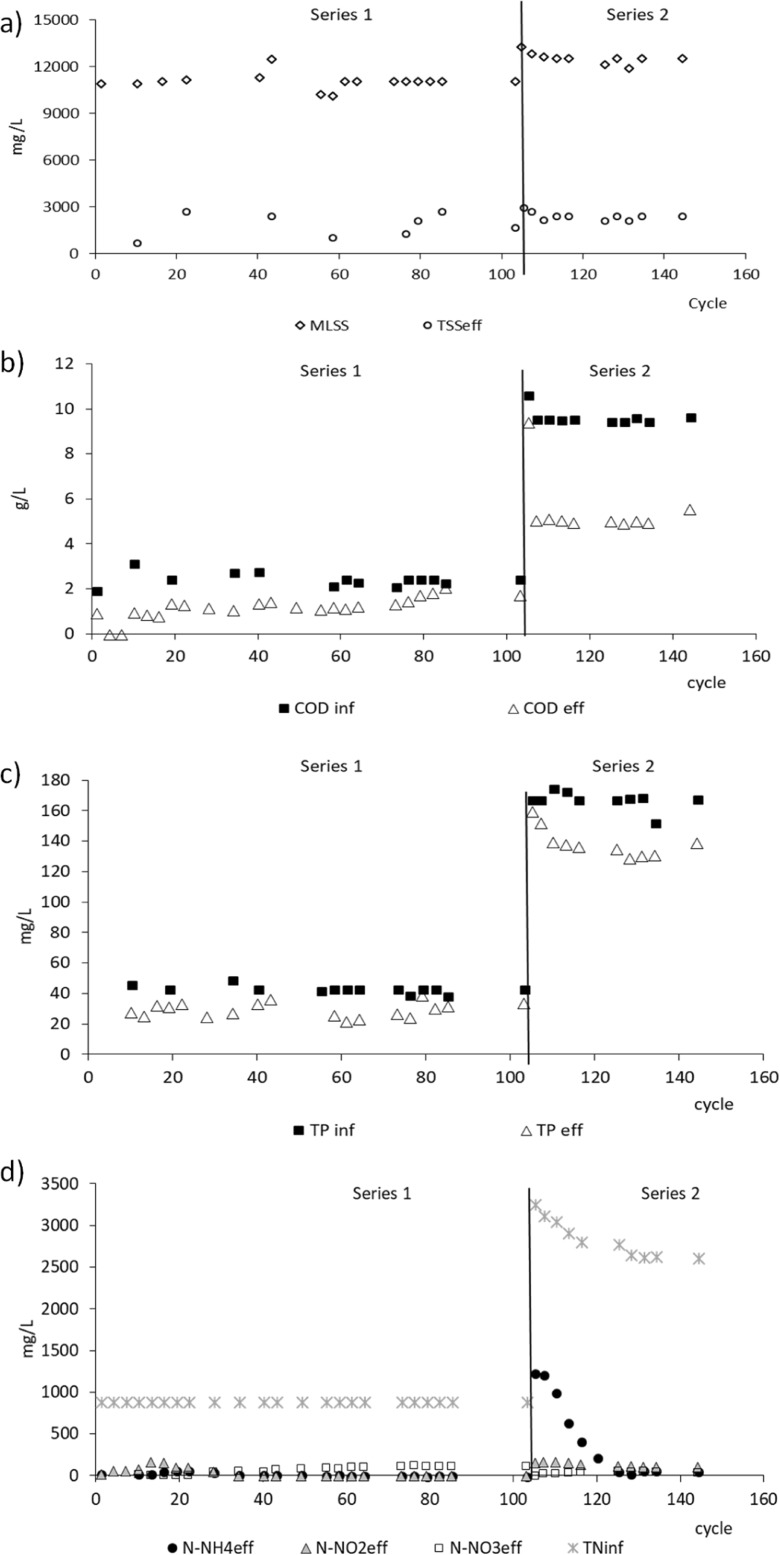
Concentration of suspended solids (MLSS) in the reactor and in the effluent (TSS_eff_) (**a**), organics in the influent (COD_inf_) and effluent (COD_eff_) (**b**), total phosphorus in the influent (TP_inf_) and the effluent (TP_eff_) (**c**), total nitrogen (TN_inf_) in the influent, ammonium nitrogen (N-NH_4eff_), and oxidized forms of nitrogen (N-NO_2eff_ nitrites, N-NO_3eff_ nitrates) in the effluent (**d**)

At both nitrogen loading rates that were tested, the average biomass concentration in the reactor was high. At 1.0 g TN/(L·day), it was 11.1 ± 0.0 g MLSS/L, of which 73.0% was the organic fraction. Increasing the share of digestate in the influent significantly increased the biomass concentration to about 13 g MLSS/L (Fig. 1a), of which 50% was the organic fraction. The observed *Y* was 0.25 and 0.16 g MLVSS/g COD at 1.0 g TN/(L·day) and 3.4 g TN/(L·day), respectively. The value of *Y* for immobilized biomass is usually low (Aspegren et al. 1998) that agrees with our results obtained for self-immobilized aerobic granular sludge. However, the values of *Y* calculated in this study depending on the organic loading rate indicate the reverse trend than that reported in the literature. For example, Bernat et al. (2013) during the treatment of synthetic wastewater observed that increase of organic loading from 0.08 g COD/(L·day) to 0.43 g COD/(L·day) increased the sludge yield coefficient from 0.56 g MLVSS/g COD to 0.76 g MLVSS/g COD. This reverse trend in our study can be explain by the fact that high nitrogen loadings favored the growth in granules of autotrophic ammonium-oxidizers that are characterized by lower growth yields (Blackburne et al. 2007; Monballiu et al. 2013) than heterotrophic microorganisms.

Digestates from methane fermentation contain a lot of suspended solids at concentrations of a few tens of g TSS/kg (Mari et al. 2014). In our experiment, the amount of TSS in the digestate was 33 g/L and the digestate contained 6.4 g TKN/L and 1.8 g N-NH_4_^+^/L. This digestate was characterized by lower pollutant concentration compared to the digestate produced after fermentation of pig slurry and maize silage at a ratio of 9:1, that was characterized by 40.7 ± 0.2 g TSS/L, 6.4 ± 0.33 g TN/L, and 4.5 ± 0.23 g N-NH_4_^+^/L (Li et al. 2018). However, the concentrations of nitrogen forms in the digestate used in this study were higher than for example values reported for digestate from fermentation of cow slurry and maize silage (from 1.9 ± 0.1 g TN/L to 2.5 ± 0.1 g TN/L and from 0.65 ± 0.05 g of N-NH_4_^+^/L to 0.84 ± 0.03 g of N-NH_4_^+^/L, Seppälä et al. 2013). The large difference between TKN and ammonium concentrations in the digestate obtained in this study indicates that most of the nitrogen was in the form of suspension, probably inorganic struvite. In anaerobic digesters, struvite is formed due to ammonium precipitating with Mg^2+^ and PO_4_^3−^ (Marti et al. 2008). Solubility of struvite depends on pH changes, temperature, and the presence of ions such as Ca^2+^. Formation of struvite is a problem in methane fermentation where pH, and ammonia and phosphate concentrations are high (Pastor et al. 2010). In the present study, part of the TSS from the influent was present in the effluent causing that less than 10% out of 2.0 g TSS/L in the effluent were in the organic fraction. Suspended solids can be efficiently removed from biologically treated digestate by, e.g., coagulation, which enabled reduction of COD that was in the form of inert organic matter to less than 40 mg/L (Zhang et al. 2011).

At a nitrogen loading rate of 1.0 g TN/(L·day), the average concentration of COD in the influent was 2.4 ± 0.1 g/L, while in the effluent, it was 1.6 ± 0.3 g/L (Fig. 1b). The concentration of soluble COD in the effluent averaged 0.347 ± 0.1 g/L, indicating that most of the COD in the effluent consisted of suspended solids. The efficiency of COD removal was 33.7 ± 14.2%. At a nitrogen loading rate of 3.4 g TN/(L·day), the concentration of COD in the influent averaged 9.5 ± 0.1 g/L, and the concentration of COD in the effluent increased significantly and averaged 5.2 ± 0.3 g/L (45.2 ± 2.2% of treatment efficiency), of which 60% was COD in the form of suspended solids. Kinetic studies performed at a nitrogen loading rate of 1.0 g TN/(L·day) showed that during the 8-h cycle, removal of COD proceeded at a rate of 1.1 g COD/(L·h) (constant of COD removal = 3.82/h), and that readily degradable organics were removed during the first hour of aeration, after which soluble COD remained at 0.75–0.80 g/L.

The total phosphorus concentration in the influent was 42.0 ± 2.0 mg/L and 165 ± 7 mg/L at the lower and higher TN loads, respectively (Fig. 1c). At both TN loadings, orthophosphates accounted for about 60% of total phosphorus, but after biological treatment, they accounted for over 90% of total phosphorus in the effluent. The removal efficiency of TP decreased from 31.7 ± 5.2% at a nitrogen loading rate of 1.0 g TN/(L·day) to 19.3 ± 3.9% at a nitrogen loading rate of 3.4 g TN/(L·day). At 1.0 g TN/(L·day) removal of orthophosphates proceeded at a rate of 2.0 mg P-PO_4_^3−^/(L·day). At the lower and higher TN loads, about 8.5 and 3.6 mg/L of phosphorus, respectively, were used for biomass synthesis and about 33 mg P/(L·day) and 76 mg P/(L·day) were removed due to enhanced phosphorus accumulation in bacterial cells.

Good phosphorus removal in biological systems requires changing anaerobic/oxic conditions. In the present study, GSBRs were constantly aerated during the cycle but large diameters of the granules ensured oxygen deficiencies in their inner parts. Studies by Wang et al. (2008) in sequencing batch reactors with an anoxic stage has shown that when the size of granules was about 950 μm, phosphorus removal efficiency was around 95%; however, a decrease in granule diameters to 620 μm significantly decreased efficiency to about 60% (Wang et al. 2009). In the present study, at 1.0 g TN/(L·day), large granules with diameters of ≥ 710 μm constituted about 29% of the biomass. That supported phosphorus removal and ensured also that the sludge had good settling properties; the SVI averaged 44.4 ± 3.9 mL/g MLSS. At nitrogen loading rate of 3.4 g TN/(L·day), the share of large granules with diameters over 2000 μm increased to 29% and SVI decreased to 41.9 ± 0.4 mL/g MLSS.

**Fig. 2:**
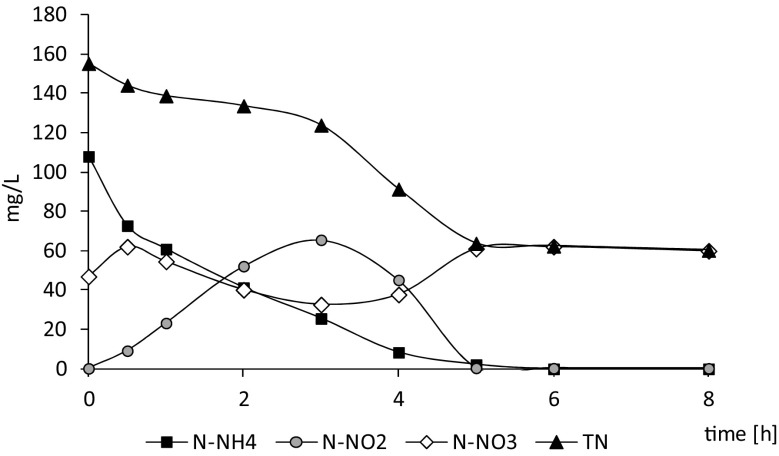
Nitrogen forms in the cycle of the reactor operated at 1.0 g TN/(L·day), N-NH_4_ – ammonium nitrogen, N-NO_2_ nitrites, N-NO_3_ nitrates, TN total nitrogen

At a nitrogen loading rate of 1.0 g TN/(L·day), the concentration of ammonium in the influent was about 392 ± 91 mg/L, while the total nitrogen concentration was 897 ± 85 mg/L, indicating that more than half of the nitrogen entering the reactor was in the form of suspended solids. Initially, the concentration of nitrogen forms in the effluent varied (Fig. 1d), but from about cycle 50 of reactor operation, effluent quality stabilized. The mean concentrations of ammonium and nitrites in the effluent were low (5.6 ± 3.8 mg/L and 0.8 ± 0.8 mg/L, respectively), while the dominant form of nitrogen was nitrates with an average concentration of 118.7 ± 5.7 mg/L. Total nitrogen in the effluent was 314 ± 88 mg/L, and over 60% was in the form of suspended solids. The average nitrogen content in the biomass was 7%, and about 12.6 mg TN/(L·cycle) was removed through biomass synthesis. The amount of nitrogen removed in denitrification was 152.8 ± 32.2 mg/L, which corresponded to 93% of the total amount of nitrogen removed. At a nitrogen loading rate of 1.0 g TN/(L·day), the rate of ammonium removal was about 16 mg N-NH_4_^+^/(L·h), while the nitrogen removal rate was about 18 mg TN/(L·h). Nitrite accumulation reached 65.6 mg/L in the third hour of the cycle. A gradual decrease in nitrite concentration from the third hour of the cycle was accompanied by an increase in nitrate concentration from the third to the fifth hour of the cycle (Fig. 2). No nitrogen removal was observed from the fifth hour of the cycle.

Increasing the share of digestate in the influent increased TN concentration to about 3200 mg/L and ammonium nitrogen to 893 ± 116 mg/L. This initially led to an increase in the ammonium nitrogen concentration in the effluent to over 1200 mg/L, but the biomass rapidly began to oxidize ammonia effectively, and after 120 cycles, the nitrogen concentration in the outflow stabilized at 55.7 ± 15.2 mg N-NH_4_/L, 115.7 ± 4.6 mg N-NO_2_/L and 53.6 ± 8.5 mg N-NO_3_/L (Fig. 1d). The increased nitrogen loading rate increased partial nitrification efficiency to 68.4 ± 3.8%. The concentrations of all forms of nitrogen were significantly higher at a nitrogen loading rate of 3.4 g TN/(L·day) than at 1.0 g TN/(L·day). The concentration of TN in the effluent at a nitrogen loading rate of 3.4 g TN/(L·day) was over 2000 mg/L, of which 90% was nitrogen in the form of suspended solids. The average nitrogen content in the biomass was 7%, and about 29.8 ± 0.3 mg TN/(L·cycle) were removed through biomass synthesis. The amount of nitrogen removed in denitrification was 256.2 ± 49.4 mg/L, which corresponded to 89% of the total amount of nitrogen removed.

At a nitrogen loading rate of 1.0 g TN/(L·day), the efficiency of ammonium removal was high 98.5 ± 1.2, while the efficiency of nitrification was 38.3 ± 9.3%. A total of 0.50 g of nitrogen was removed per day per liter of reactor volume. The denitrification efficiency was 55.5 ± 6.6% and the overall efficiency of nitrogen removal, calculated based on influent and effluent concentrations, was 64.9 ± 9.8%. At a nitrogen loading rate of 3.4 g TN/(L·day), the efficiencies of ammonium removal and nitrification were 93.6 ± 2.0 and 12.6 ± 1.4%, respectively. The overall efficiency of N removal was 30.2 ± 2.6%, and the amount of nitrogen removed was 0.86 g N/(L·day). Higher removal of nitrogen at a 3.4 g TN/(L·day) probably resulted from the higher availability of substrates for denitrification. The amount of oxidized nitrogen forms at the end of reactor cycle at higher nitrogen loading rate was about 170 mg NO_*x*_/L while the concentration of oxidized nitrogen forms at the lower nitrogen loading rate was about 120 mg/L. High concentration of NO_*x*_ combined with the higher availability of organics at a nitrogen loading rate of 3.4 g TN/(L·day) resulted in higher overall nitrogen removal.

Removal of ammonium from digestate is crucial because it is the most problematic form of nitrogen, not only causing undesirable odors, but also easily migrating in soil, leading to eutrophication of water bodies. Overuse of digestate as a soil fertilizer can not only worsen the quality of soils but also pose a risk to consumers of agricultural products due to the accumulation of nitrogen compounds, especially nitrites and nitrates, in vegetables (Larsen et al. 2014; Koca et al. 2016). In the present study, at a nitrogen loading rate of 1.0 g/(L·day), the main nitrification product was nitrate while at a higher nitrogen loading rate, partial nitrification predominated. The fact that nitrite concentration significantly increased the total amount of NO_*x*_ in the effluent after increasing ammonia load suggests that nitrite oxidation was inhibited by free ammonia (FA). The concentration of FA at the beginning of the reactor cycle was 244.9 ± 26.4 mg/L. The FA inhibition threshold is 10–150 mg NH_4_^+^-N/L and 0.1–4.0 mg NH_4_^+^-N/L for *Nitrosomonas* sp. and *Nitrobacter* sp., respectively (Anthonisen et al. 1976; Bae et al. 2001), which were the predominant autotrophic nitrifiers in aerobic granules. The FA concentration observed in the experiment is higher than the inhibition threshold for both genera, which can explain the observed ammonium and nitrite accumulation in the effluent.

An accumulation of nitrites was previously observed during the treatment of the liquid phase of digestate from anaerobic stabilization of pig manure in an intermittently aerated sequencing batch reactor with activated sludge (Zhang et al. 2011). At a mean nitrogen load of 0.38 g N/(L·day) and an organics load of 1.15 g COD/(L·day), the partial nitrification efficiency was about 70%. In our study, the application of about three times higher nitrogen and organics loads still enabled nitrification to nitrates indicating better resilience of aerobic granules to high pollutant loading than activated sludge. Nonetheless, the composition of the biologically treated digestate indicates that it would still introduce large quantities of nitrite and nitrate to the environment.

In the present study, at a lower nitrogen loading rate of 1.0 g/(L·day), the quality of biologically treated digestate was better—ammonium was fully removed, and nitrates were the main nitrification product. On the other hand, at the nitrogen loading rate of 3.4 g/(L·day), the overall amount of nitrogen removed was higher and reached 0.86 g/(L·day). By extrapolating from our results, it can be assumed that further treatment of digestate ensuring the complete removal of nitrogen present in suspended solids from the effluent (e.g., due to microfiltration) would decrease the load of nitrogen from 0.31 g TN/(L·day) and 2.22 g TN/(L·day) to 0.12 g TN/(L·day) and 0.22 g TN/(L·day) at nitrogen loading rates of 1.0 g/(L·day) and 3.4 g/(L·day), respectively. Assuming complete removal of NO_*x*_ from the effluent in the post-denitrification step (e.g., during storage and based on organics present in the biologically treated digestate) further decrease of the load of nitrogen to 0.006 g TN/(L·day) and 0.056 g TN/(L·day), respectively, would be observed.

To investigate the microbial structure of digestate and aerobic granules, next-generation sequencing was performed targeting the 16S rDNA gene. High diversity and stability of investigated communities was observed, and the Chao 1 estimator, Shannon-Wiener index, and Evenness index were 56,938.7, 6.8 ± 0.0, and 0.75 for digestate, and 59,647.7, 7.2 ± 0.0 and 0.8 for aerobic granular sludge, respectively.

**Fig. 3:**
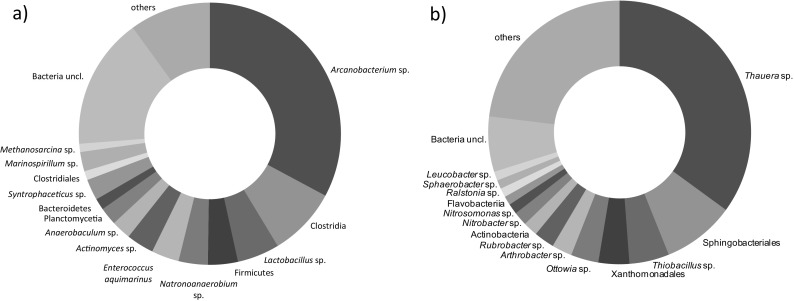
Bacterial taxa in digestate (**a**) and aerobic granular sludge from the reactor treating digestate at 1.0 g TN/(L·day) (**b**)

In the digestate, the phylum Actinobacteria predominated, especially *Arcanobacterium* sp.; this phylum comprised nearly 33% of the identified sequences (Fig. 3a). These bacteria are facultative anaerobes that can degrade sugars such as glucose and lactose (Collins et al. 1982). Microorganisms belonging to Firmicutes comprised about 25% of the bacteria, and the most numerous were genera belonging to the classes Clostridia and Bacilli. Microorganisms belonging to the class Clostridia can efficiently catabolize proteins and hydrolyze cellulose, which supports degradation of plant substrates and animal manure in the feedstock (Lu et al. 2016). Within Clostridia, the most numerous were *Natronoanaerobium* sp. (3.6%) and *Syntrophaceticus* sp. (2.6%). *Syntrophaceticus* sp. are syntrophic acetate-oxidizing bacteria that cooperate with hydrogen-utilizing methanogens (Westerholm et al. 2010). Within Bacilli, the most numerous were *Enterococcus aquimarinus* (3.44%) and *Lactobacillus* sp. (5.2%; *L*. *kefiranofaciens* 1.4%), which are able to produce short-chain acids from many substrates, such as glucose, fructose, mannose, or starch (Švec 2005). Over 2% of the sequences belonged to Planctomycetia, *Anaerobaculum* sp. (class Synergistia), and *Marinospirillum* sp. Among the methanogens, *Methanosarcina* sp., which are able to produce methane using all three known metabolic pathways, i.e., from carbon dioxide and hydrogen gas, by utilizing acetate and by metabolizing methylated one-carbon compounds through methylotrophic methanogenesis were identified in relatively large amounts (1%). Such a versatility resulted in stable methane fermentation that was observed in the digesters.

In aerobic granules treating digestate, the phylum Proteobacteria predominated and consisted of alpha-, beta-, and gammaproteobacteria. Most of these were members of the class betaproteobacteria. At high nitrogen and organics loads, bacteria with various N metabolism routes comprised over 50% of the biomass of the aerobic granules.

In conventional treatment systems, biological ammonium removal takes place via autotrophic nitrification and heterotrophic denitrification. In such systems, low nitrification rates and deterioration of nitrifiers’ activity at high loads of ammonium and organics make long hydraulic retention times or large reactor volumes necessary to completely remove NH_4_^+^. Over the last decades, other bioprocesses for ammonium removal from municipal and domestic wastewaters have been developed, including shortcut nitrification and denitrification, aerobic deammonification, complete autotrophic nitrogen removal over nitrite, oxygen-limited nitrification, and denitrification or anaerobic ammonium oxidation (Shoda and Ishikawa 2014). All of these, however, are based on ammonium oxidation by autotrophic microorganisms; thus, their application for the treatment of wastewater with high loads of both ammonium and organics is very limited.

Our data indicate that aerobic granular sludge exposed to the high organics and nitrogen loading present in digestate was able to successfully remove ammonium. The microbial composition of the granular biomass indicates that heterotrophic nitrification was the main pathway of ammonium removal.

The advantages of heterotrophic nitrification are that nitrification and denitrification take place simultaneously at high organic loadings, and this simultaneous nitrification/denitrification helps to avoid acidification of the reactor (Chen and Ni 2011; Su et al. 2015). Many microorganisms isolated from wastewater treatment systems, including *Paracoccus denitrificans*, *Alcaligenes faecalis*, *Pseudomonas* sp., *Thauera* sp., *Rhodoferax ferrireducens*, *Rhodococcus* sp. CPZ 24, *Bacillus* sp., *Acinetobacter* sp., *Diaphorobacter* sp., *Marinobacter* sp., and *Microvirgula aerodenitrificans*, have been capable of heterotrophic nitrification and aerobic denitrification (Takenaka et al. 2007; Yao et al. 2013; Shoda and Ishikawa 2014; Scholten et al. 1999; Khanichaidecha et al. 2018). Pure microbial cultures of heterotrophic nitrifiers/aerobic denitrifiers were used for efficient treatment of wastewater from pig farms (Joo et al. 2006) or saline wastewater (Duan et al. 2015). Lengthening of anoxic periods in the cycle of a sequencing batch reactor with aerobic granules operated at a high nitrogen load caused autotrophic nitrifiers to disappear from the biomass and ammonium removal take place via heterotrophic nitrification by *Pseudomonas* sp. and *Paracoccus* sp. (Cydzik-Kwiatkowska 2015).

In the present study, next-generation sequencing was used, which showed that *Thauera* sp. comprised about 35% of all obtained sequences and consisted mostly of *Thauera terpenica* (over 27% of all identified sequences). *T*. *terpenica* can utilize bicyclic eucalyptol and monocyclic monoterpene alkenes and mineralize them to carbon dioxide while nitrate is reduced to dinitrogen (Foss and Harder 1998). Monoterpenes are very common in plants and were probably present in the fermented plant feedstock and then in the treated digestate, favoring the growth of *T*. *terpenica* in the present study. Some small amounts (less than 0.5%) of other heterotrophic nitrifiers/autotrophic denitrifiers such as *Pseudomonas* sp., *Paracoccus* sp., *Rhodococcus* sp., *Bacillus* sp., and *Acinetobacter* sp. were also present in granules. The heterotrophic nitrification activity of *Thauera* sp. was supported by autotrophic nitrifiers. In the biomass, *Nitrosomonas* sp. (1.3%) and *Nitrobacter* sp. (1.7%) were present, which are well recognized as microorganisms that tolerate high nitrogen loading conditions (24 g/day) (Dytczak et al. 2008).

*Thauera* sp. are capable of aerobic denitrification, which in the aerated reactors that were used in this study, gives them an advantage over other denitrifiers that prefer anoxic conditions. *Thauera* sp. may store PHB (Scholten et al. 1999) and produce EPS, which assists in formation of microbial aggregates during combined nitritation, anammox, and denitrification processes for nitrogen removal from ammonium-rich wastewater (Langone et al. 2014). Microorganisms belonging to *Thauera* sp. reduce oxidized forms of nitrogen, but the compound that is reduced depends on the particular species. Some *Thauera* strains can rapidly express all their denitrification genes, so that there is no detectable nitrite accumulation, whereas other strains progressively activate different denitrification genes. *T*. *terpenica*, which predominated in our study, is one of the strains that progressively activates these genes, and Liu et al. (2013) reported that transcripts of the nitrate reductase gene were not detected until all available nitrate was consumed. Our kinetic studies show that denitrification of both nitrites and nitrates occurred during the cycle, indicating that other denitrifying bacteria simultaneously supported the activity of *Thauera terpenica*.

Denitrifying taxa that were abundant in the granules were Xanthomonadales, *Thiobacillus* sp. and *Ottowia* sp. *Ottowia* sp. has been identified as an efficient denitrifier during the treatment of high-nitrogen piggery wastewater with a low C/N ratio in a distributed-inflow biological reactor (Zhong et al. (2016), while *Thiobacillus* sp. completely reduced nitrate, simultaneously producing extracellular polymeric substances (lipopolysaccharides) (Gehrke et al. 1998). Production of EPS is advantageous because EPS mediate contact between bacterial cells and support granule formation and bacterium-substratum interactions (Hosono et al. 2015).

The phyla Bacteroidetes and Actinobacteria were also important components of the biomass. Within Actinobacteria, the most numerous were members of the genera *Rubrobacter* (2.5%) and *Arthrobacter* (2.5%), while the members of Bacteroidetes mostly belonged to Sphingobacteriales (about 9% of all sequences). Species within Sphingobacteria have been observed in microbial communities that decomposed lignocellulose under thermophilic and high-solid conditions (Yu et al. 2015), lignin in a thermophilic environment (Taylor et al. 2012), or hemicellulose at 25 °C (Jiménez et al. 2015; Jiménez et al. 2014). The results of our study indicate that Sphingobacteriales may have been responsible for decomposition of the complex organic material that was present in the form of TSS in the fermented digestate.

In the aerobic granules and digestate, about 7 and 16% of sequences, respectively were only classified to the kingdom Bacteria indicating the high diversity of yet unknown species.

## Conclusions

The use of aerobic granular sludge allowed efficient removal of soluble ammonium nitrogen from digestate. At the lower nitrogen loading rate of 1.0 g/(L·day), the quality of biologically treated digestate was better, but at the nitrogen loading rate of 3.4 g/(L·day), the overall amount of nitrogen removed was higher. More than 50% of all bacteria in the aerobic granules were able to metabolize nitrogen. The predominant genera (35%) was *Thauera*, a denitrifier capable of heterotrophic nitrification, which indicated that stable ammonium removal was achieved because of synergistic effects of autotrophic and heterotrophic nitrifier activity. The biologically treated digestate contained large amounts of inert suspended solids with a large fraction of total nitrogen and a high concentration of oxidized nitrogen forms; if required, post-denitrification and total suspended solid separation (e.g., membrane filtration) can be used to enable full nitrogen removal. Alternatively, the digestate can be separated into solid and liquid fractions, and the liquid fraction could be biologically treated.
